# Respiratory problems in early childhood and molar-incisor hypomineralization: systematic review and meta-analysis

**DOI:** 10.1007/s10266-026-01321-7

**Published:** 2026-02-10

**Authors:** Ronney Brandão Osterno, Lara Carvalho Costa, Francisco Wilker Mustafa Gomes Muniz, Marta Maria Alves Pereira, Marcoeli Silva de Moura, Cacilda Castelo Branco Lima, Lúcia de Fátima Almeida de Deus Moura, Marina de Deus Moura Lima

**Affiliations:** 1 Department of Pathology and Dental Clinic, Federal University of Piauí, Teresina, Piauí Brazil; 2 Department of Periodontology, Federal University of Pelotas, Pelotas, Rio Grande do Sul, Brazil; 3 Postgraduate Program in Dentistry, Federal University of Piauí, Teresina, Piauí Brazil

**Keywords:** Respiratory infections, Respiratory tract disease, Developmental defects of enamel, Molar hypomineralization, Systematic review

## Abstract

**Purpose:**

To evaluate the association between early childhood respiratory problems and Molar-Incisor Hypomineralization (MIH).

**Methods:**

Observational studies assessing the relationship between early respiratory diseases and MIH were included. Two independent reviewers conducted comprehensive searches in MEDLINE-PubMed, Scopus, EMBASE, Web of Science, Google Scholar^®^, and OpenGrey^®^. This systematic review followed PRISMA guidelines and was registered in the PROSPERO database (CRD42022320707). Risk of bias was assessed using the Joanna Briggs Institute (JBI) checklist for cross-sectional studies and the Newcastle–Ottawa Scale for case–control and cohort studies. The certainty of the evidence was rated using the GRADE framework. Meta-analyses were performed to explore associations between respiratory conditions and MIH (significance level p < 0.05).

**Results:**

From 1228 identified articles, 27 met the inclusion criteria, and 12 were included in four meta-analyses. Most cross-sectional and cohort studies showed low risk of bias, while case–control studies showed moderate risk. Quantitative synthesis was feasible only for asthma and pneumonia. In adjusted analyses, asthma was not significantly associated with MIH (OR 1.93; 95% CI 0.60–6.20), with substantial heterogeneity across studies. Pneumonia showed a positive overall association with MIH (OR 3.33; 95% CI 1.05–10.51). However, the certainty of evidence for all associations was considered very low.

**Conclusion:**

Early childhood respiratory conditions may be associated with molar–incisor hypomineralization, but the available evidence is inconsistent and of very low certainty, precluding causal inference.

**Supplementary Information:**

The online version contains supplementary material available at https://doi.org/10.1007/s10266-026-01321-7.

## Introduction

Molar-incisor hypomineralization (MIH) is a qualitative developmental defect of dental enamel characterized by the involvement of at least one permanent first molar, with potential extension to the incisors as well [1]. The estimated global prevalence of MIH is approximately 14.3% [2].

Clinically, MIH is identified by demarcated opacities that range in color from white to brown, depending on the severity of the condition, and is frequently associated with an increased risk of dental caries [3, 4]. The hypomineralized enamel exhibits a reduced mineral content, rendering it brittle and prone to post-eruptive structural breakdown. Consequently, restorative failures are common, often leading to multiple dental reinterventions over time [3, 5].

Indeed, MIH poses a significant challenge in pediatric dentistry [6, 7]. The exposure of dentin may provoke pulpal inflammation, which complicates anesthesia and may elicit anxiety and fear in affected children [6, 7]. Moreover, MIH has been shown to adversely affect oral health-related quality of life (OHRQoL) in pediatric patients [8–11].

The etiology of MIH is multifactorial, involving environmental, genetic, and epigenetic influences [12]. Among the environmental contributors, respiratory illnesses during early childhood are noteworthy due to their high prevalence and potential association with episodes of hypoventilation and hypoxia [13–15]. These conditions may induce respiratory acidosis and reduce tissue oxygenation, thereby disrupting the pH balance of the enamel matrix. Such disturbances can interfere with the maturation phase of amelogenesis by inhibiting enzymatic activity and altering hydroxyapatite crystal growth [15–17]. Therefore, adverse events occurring during early childhood may contribute to enamel developmental defects, including MIH [5].

Although previous studies have investigated the relationship between early childhood respiratory diseases and dental hypomineralization [12, 18], the findings remain inconclusive [19, 20]. Accordingly, the objective of this systematic review is to determine whether a significant association exists between respiratory diseases in early childhood and the development of MIH.

## Materials and methods

This systematic review addressed the research question: “Is there an association between respiratory diseases in early childhood and molar incisor hypomineralization (MIH)?” Early childhood was defined as the period from birth to 3 years of age, corresponding to the enamel apposition and maturation phases of first permanent molars and incisors [4–6]. The review was conducted in accordance with the Preferred Reporting Items for Systematic Reviews and Meta-Analyses (PRISMA) Statement guidelines [21] (see Supplementary Material) and was prospectively registered in the International Prospective Register of Systematic Reviews (PROSPERO) under registration number CRD42022320707.

### Eligibility criteria

The PECO framework guided the formulation of the research question: P (population): dentate individuals with no age restriction; E (exposure): individuals with respiratory disorders diagnosed either by a physician or reported by parents; C (comparison): individuals without respiratory disorders; O (outcome): MIH diagnosed by an oral health professional.

Eligible study designs included cross-sectional, case–control, and cohort studies (both prospective and retrospective) that investigated the association between respiratory conditions in early childhood and MIH. Exclusion criteria comprised studies lacking a comparison group, those failing to differentiate MIH from other enamel developmental defects, pilot studies, and those with incomplete data.

### Search strategy

A comprehensive literature search was conducted in July, 2025, across four databases—Medline/PubMed (National Library of Medicine), Scopus (Elsevier), EMBASE (Elsevier), and Web of Science (Clarivate Analytics)—without restrictions on language or publication year. The PubMed search strategy is detailed below and was adapted accordingly for the remaining databases. Reference management and duplicate removal were performed using EndNote Web^®^ and Rayyan^®^ software, both automatically and manually.

#### PubMed Search Strategy

#1 (EXPOSURE)—Respiratory Tract Diseases[Mesh term] OR Respiratory Tract Diseases[Text word] OR Disease, Respiratory Tract[Text word] OR Diseases, Respiratory Tract[Text word] OR Respiratory Tract Disease[Text word] OR Tract Disease, Respiratory[Text word] OR Tract Diseases, Respiratory[Text word] OR Respiratory Hypersensitivity[Mesh Term] or Respiratory Hypersensitivity[Text word] OR Airway Hyper-Responsiveness[Text word] OR Airway Hyper Responsiveness[Text word] OR Hyper-Responsiveness, Airway[Text word] OR Hypersensitivity, Respiratory[Text word] OR Hypersensitivities, Respiratory[Text word] OR Respiratory Hypersensitivities[Text word] OR Asthma[Mesh term] OR Asthma[Text word] OR Asthmas[Text word] OR Bronchial Asthma[Text word] OR Asthma, Bronchial OR Rhinitis[Mesh terms] OR Rhinitis [Text word] OR Rhinitides[Text word] OR Nasal Catarrh[Text word] OR Catarrh, Nasal[Text word] OR Catarrhs, Nasal[Text word] OR Nasal Catarrhs[Text word] OR Rhinitis, Allergic[Mesh Term] OR Rhinitis, Allergic[Text word] OR Allergic Rhinitides[Text word] OR Rhinitides, Allergic[Text word] OR Allergic Rhinitis[Text word] OR Respiratory Tract Infections[Mesh term] OR Respiratory Tract Infections[Text word] OR Infection, Respiratory Tract[Text word] OR Respiratory Tract Infection[Text word] OR Infections, Respiratory[Text word] OR Infections, Respiratory Tract[Text word] OR Respiratory Infections[Text word] OR Upper Respiratory Tract Infections[Text word] OR Upper Respiratory Infections[Text word] OR Upper Respiratory Tract Infection[Text word] OR Infections, Upper Respiratory Tract[Text word] OR Infections, Upper Respiratory[Text word] OR Respiratory Infection, Upper[Text word] OR Bronchitis[Mesh Term] OR Bronchitis[Text word] OR Bronchitides[Text word] OR Pneumonia[Mesh term] OR Pneumonia[Text word] OR Pneumonias[Text word] OR Lobar Pneumonia[Text word] OR Lobar Pneumonias[Text word] OR Pneumonias, Lobar[Text word] OR Pneumonia, Lobar[Text word] OR Experimental Lung Inflammation[Text word] OR Experimental Lung Inflammations[Text word] OR Inflammation, Experimental Lung[Text word] OR Lung Inflammation, Experimental[Text word] OR Lung Inflammations, Experimental[Text word] OR Pneumonitis[Text word] OR Pneumonitides[Text word] OR Pulmonary Inflammation[Text word] OR Inflammation, Pulmonary[Text word] OR Inflammations, Pulmonary[Text word] OR Pulmonary Inflammations[Text word] OR Lung Inflammation[Text word] OR Inflammation, Lung[Text word] OR Inflammations, Lung[Text word] OR Lung Inflammations[Text word] OR Pharyngitis[Mesh terms] OR Pharyngitis[Text word] OR Pharyngitides[Text word] OR Sore Throat[Text word] OR Sore Throats[Text word] OR Throat, Sore[Text word].

#2 (OUTCOME)—Dental Enamel Hypoplasia[Mesh terms] OR Dental Enamel Hypoplasia[Text word]; Agenesis, Enamel[Text word] OR Enamel Hypoplasia[Text word] OR Hypoplasias, Enamel[Text word] OR Molar Incisor Hypomineralization[Text word] OR Hypomineralization[Text word] OR Hypomineralisation[Text word] OR Hypomineralised[Text word] OR Hypomineralized second primary molar[Text word] OR Hypomineralised Second Primary Molar[Text word] OR Molar Incisor Hypomineralisation[Text word] OR Deciduous Molar Hypomineralization[Text word] OR Deciduous Molar Hypomineralisation[Text word] OR Molar Incisor[Text word] OR Amelogenesis Imperfecta[Text word] OR enamel defects[Text word] OR cheese molars[Text word] OR enamel opacities[Text word] OR Hypoplastic Enamel[Text word] OR Enamel, Hypoplastic[Text word] OR Enamel Hypoplasia, Dental[Text word] OR Hypoplasia, Dental Enamel[Text word] OR Enamel Agenesis[Text word] OR Ageneses, Enamel[Text word] OR Enamel Ageneses[Text word] OR Enamel Hypoplasias[Text word] OR Hypoplasia, Enamel[Text word] OR Hypomineralization[Text word] OR Congenital Enamel Hypoplasia[Text word] OR hypomineralized[Text word] OR hypomineralisation[Text word] OR Tooth Hypomineralization[Text word] OR Hypomineralization, Tooth[Text word] OR Hypomineralizations, Tooth[Text word] OR Tooth Hypomineralizations[Text word] OR non-fluoride enamel opacities[Text word] OR internal enamel hypoplasia[Text word] OR demarcated opacities[Text word] OR idiopathic enamel opacities[Text word].

#3—#1 AND #2

### Study selection process

Two independent reviewers (R.B.O. and L.C.C.) conducted study selection, with a third reviewer (M.D.M.L.) resolving any disagreements. In the initial screening phase, titles and abstracts were assessed based on the predefined eligibility criteria, yielding a Kappa coefficient of 0.80, indicative of substantial inter-rater agreement. In the subsequent phase, full texts of the selected articles were assessed (Kappa = 1.0). To ensure a comprehensive search, an additional hand search of relevant journals from the last 5 years was performed, targeting Pediatric Dentistry, European Journal of Pediatric Dentistry, and International Journal of Pediatric Dentistry, based on journal relevance and citation frequency in pediatric dentistry, according to the Web of Science website. Reference lists of included studies were also manually screened for potentially eligible articles not retrieved in the database search.

Gray literature was explored using a tailored manual search on OpenGrey and the first 300 articles on Google Scholar^®^, employing the following descriptors: “Molar incisor hypomineralization”, “Molar incisor hypomineralisation”, “Respiratory tract disease”, “Asthma”, “Bronchitis”, and “Pneumonia”.

### Data extraction

Two reviewers (R.B.O. and L.C.C.) independently extracted data and organized it using a customized Microsoft Excel^®^ spreadsheet (Version 2014, USA) developed specifically for this study. Extracted data included: first author and year of publication, country of sample origin, language, study design, sample size (n), participant age and gender, outcome assessment and diagnostic criteria for MIH, funding sources, conflict of interest disclosures, and principal findings.

At this stage, corresponding authors were contacted via email to obtain clarifications or missing information when necessary.

### Risk of bias assessment

The risk of bias in the included studies was assessed independently by two reviewers (R.B.O. and L.C.C.), with a third reviewer (M.D.M.L.) available for adjudication. Where, cross-sectional studies were appraised using the Joanna Briggs Institute (JBI) Critical Appraisal Checklist [22], comprising eight questions with responses categorized as “yes”, “no”, “unclear”, or “not applicable”.

Studies scoring over 70% “yes” responses were considered low risk of bias; 50–69%, moderate risk; and < 49%, high risk [23]. Case–control and cohort studies were evaluated using the Newcastle–Ottawa Scale (NOS) [24], which includes three domains: Selection, Comparability, and Exposure. Studies receiving 7–9 stars were deemed low risk; 4–6 stars, moderate risk; and 0–3 stars, high risk [23, 25].

### Meta-analyses

Meta-analyses were conducted only for outcomes with at least four contributing studies and acceptable clinical and methodological comparability. Outcomes represented by less than four studies and/or exhibiting extreme heterogeneity were not quantitatively pooled and were instead summarized narratively. For unadjusted analyses, odds ratios (ORs) and their corresponding 95% confidence intervals (CIs) were computed for each included study. As for adjusted analyses, inclusion required adjustment for at least one confounding variable in addition to the respiratory condition. The natural logarithm (log) of the OR was calculated for studies providing adjusted estimates. No restrictions were placed on the type of variable adjusted for. Subgroup meta-analyses were conducted according to study design.

Preplanned subgroup analyses according to respiratory diagnosis subtype, exposure source (medical records versus parental recall), and disease severity were explored descriptively; however, subgroup analyses were not performed quantitatively for these variables due to the insufficient number of studies.

A significance threshold of 5% was adopted. Heterogeneity among studies was evaluated using Cochran’s Q test and quantified with the I^2^ statistic. Whenever quantitative synthesis was deemed methodologically appropriate, random-effects models were applied due to anticipated clinical and methodological heterogeneity [26]. RevMan^®^ software (Cochrane Collaboration, version 5.0, United Kingdom) was used for the analyses.

### Certainty of evidence

The certainty of the evidence was assessed based on the Grading of Recommendations Assessment, Development and Evaluation (GRADE) approach [27]. Independent analyses were conducted for each respiratory condition in relation to MIH. A summary of findings was compiled using the GRADEpro GDT software. Statistical imprecision was judged based on the width of confidence intervals, with extremely wide intervals interpreted as indicative of substantial uncertainty and contributing to downgrading for imprecision within the GRADE framework.

## Results

**Fig. 1:**
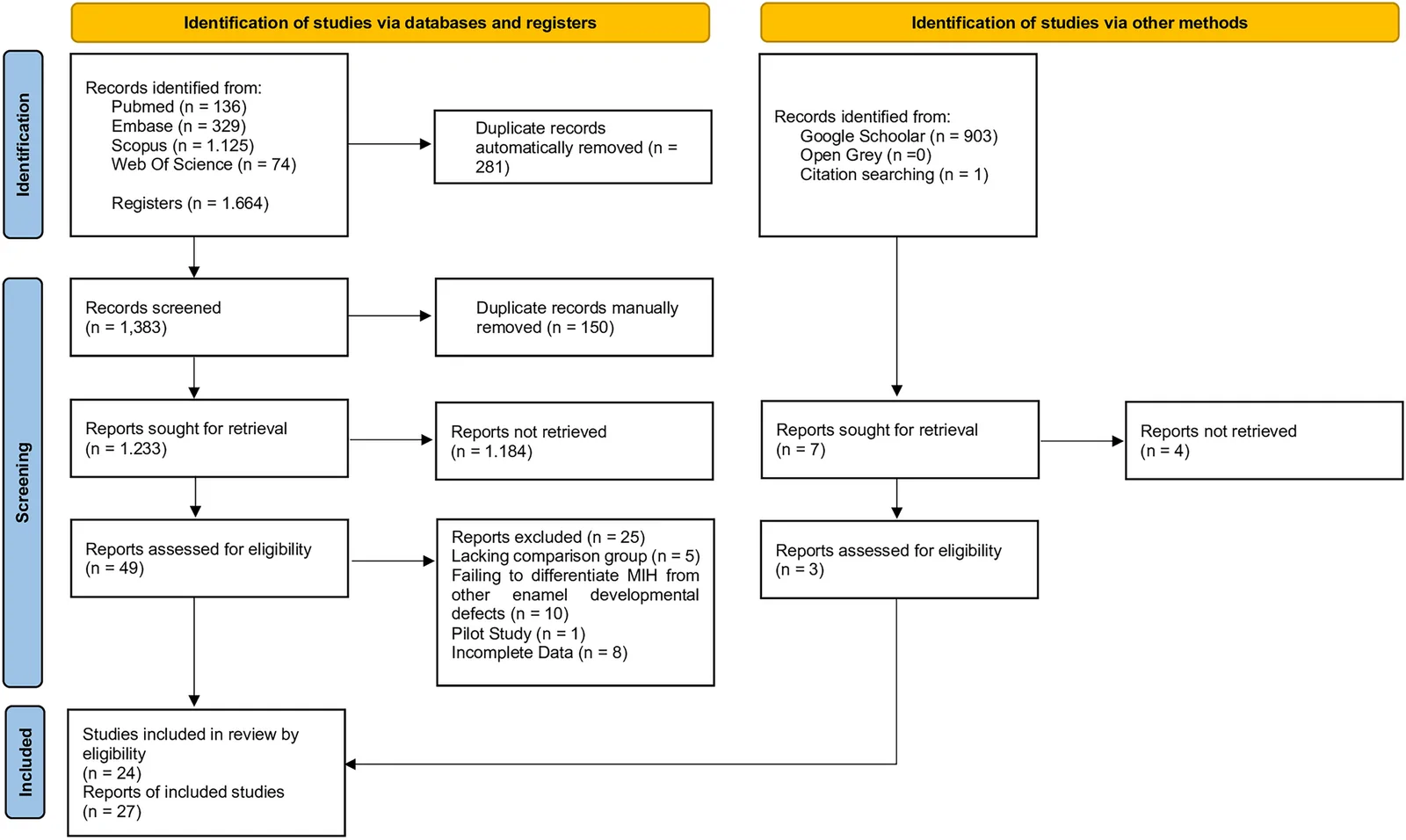
Flowchart of study inclusion based on PRISMA guidelines

A total of 1228 potentially relevant articles were initially identified. Of these, 209 were automatically excluded by EndNote software, and an additional 130 were manually excluded in Rayyan due to duplication. After removing duplicates, 889 studies remained for title and abstract screening. Of these, 43 articles were deemed eligible for full-text assessment, resulting in the exclusion of 19 studies. Additionally, three relevant studies were identified through manual search and gray literature, culminating in 27 studies included in this systematic review (the study selection process is summarized in Fig. 1).

### Characteristics of included studies

All included studies were published between 2001 and 2022, and all were written in English. The number of examiners ranged from one to nine. Sample sizes varied from 45 to 4,049 participants, with age ranges between six and 28 years; exposure to respiratory conditions was retrospectively assessed for early childhood (birth to 3 years of age). The majority of studies were conducted in Brazil [4, 28–32], and most employed a cross-sectional design [4, 8, 12, 17, 19, 20, 28, 29, 32–43].

For the diagnosis of Molar-Incisor Hypomineralization (MIH), the diagnostic criteria proposed by the European Academy of Paediatric Dentistry (EAPD) were most commonly used [4, 8, 12, 17, 19, 20, 28–32, 34–41, 44–47]. Regarding exposure assessment, the majority of studies utilized structured questionnaires [4, 8, 12, 17, 19, 28–30, 32–34, 36, 39, 41, 43, 45–48]. Dental examinations were carried out in schools [4, 8, 12, 19, 28, 29, 32, 41, 43, 47], dental clinics (both public and private) [17, 20, 35, 37, 38, 40, 42, 44–46], or university settings [30, 36, 39].

A comprehensive overview of the included studies is provided in Table 1, detailing their main characteristics. All studies assessed the association between MIH and multiple etiological factors. Specifically: 19 studies investigated lower respiratory tract infections (e.g., chest infections, bronchitis, bronchiolitis, pneumonia) [4, 8, 17, 19, 20, 28, 30, 32, 33, 36, 37, 39, 41, 42, 44, 45, 47, 48]; 16 studies examined asthma as an exposure variable [4, 8, 19, 30, 32–34, 36, 37, 39–41, 43–45, 47]; 15 studies explored upper respiratory tract infections (e.g., tonsillitis, pharyngitis, laryngitis, rhinitis) [8, 17, 19, 20, 29–31, 33, 36, 37, 41, 42, 44, 47, 48]. Moreover, 3 studies reported an association between respiratory problems and MIH but did not specify which respiratory conditions were assessed [12, 31, 46].

**Table 1:** Comprehensive overview of the included studies

Authors (year); country	Study type	Sample (age)	MIH index	Respiratory diagnosis	Main findings
Jälevik et al. (2001); Switzerland	Cross-sectional	516 (8 y)	mod. DDE	Questionnaire	MIH assoc. w/asthma ( OR 24), pneumonia ( OR 2.5)
Beentjes et al. (2002); Netherlands	Case–control	45 (9.9 y)	NR	Questionnaire	Assoc. w/otitis, pneumonia, fever
Muratbegovic et al. (2007); Bosnia	Cross-sectional	560 (12 y)	EAPD	Questionnaire	No assoc. w/tonsillitis, bronchitis, pneumonia
Lygidakis et al. (2008); Greece	Case–control	945 (≤ 12 y)	EAPD	Records + Q	Assoc. w/upper/lower resp. diseases
Wogelius et al. (2010); Denmark	Cross-sectional	745 (6–8 y)	EAPD	Medical	No assoc. w/asthma
Souza et al. (2012); Brazil	Cross-sectional	903 (6–12 y)	EAPD	Questionnaire	Rural group: assoc. w/fever, throat inf.
Sönmez et al. (2013); Turkey	Cross-sectional	4049 (7–12 y)	EAPD	Questionnaire	Assoc. w/pneumonia, fever; not asthma
Souza et al. (2013); Brazil	Cross-sectional	1151 (7–12 y)	EAPD	Questionnaire	No assoc. w/pneumonia, bronchitis
Allazzam et al. (2014); Saudi Arabia	Cross-sectional	267 (8–12 y)	EAPD	Questionnaire	Assoc. w/tonsillitis, asthma, antibiotics
Kühnisch et al. (2014); Germany	Cross-sectional	692 (10 y)	EAPD	Questionnaire	Assoc. w/early resp. disease (OR 2.48)
Pitiphat et al. (2014); Thailand	Cross-sectional	420 (7–8 y)	EAPD	Questionnaire	Assoc. w/asthma, pneumonia, tonsillitis
Tourino et al. (2016); Brazil	Cross-sectional	1181 (8–9 y)	EAPD	Questionnaire	Assoc. w/pneumonia, asthma, antibiotics
Wuollet et al. (2016); Finland	Cross-sectional	287 (7–12 y)	EAPD	Medical	Assoc. w/otitis, antibiotics
Hernandez et al. (2018); Spain	Cross-sectional	705 (6–14 y)	EAPD	Questionnaire	Assoc. w/pneumonia, asthma, fever
Portella et al. (2018); Brazil	Case–control	93 (6–13 y)	EAPD	Questionnaire	No assoc.
Kılınç et al. (2019); Turkey	Cross-sectional	1237 (9–10 y)	EAPD	Questionnaire	Assoc. w/fever, asthma
Mejía et al. (2019); Colombia	Cross-sectional	1075 (6–15 y)	EAPD	Questionnaire	Assoc. w/early resp. problems (OR 2.48)
Flexeder et al. (2020); Germany	Cohort	730 (10–15 y)	EAPD	Questionnaire	Assoc. at 15 y (OR 2.56), not at 10 y
Hernandez et al. (2020); Spain	Case–control	102 (8–12 y)	EAPD	Medical	Assoc. w/asthma (OR 1.71)
Lee et al. (2020); S. Korea	Case–control	1191 (6–13 y)	EAPD	Questionnaire	Assoc. w/resp. infections (OR 1.33)
Wogelius et al. (2020); Denmark	Cross-sectional	1837 (9 y)	EAPD	Medical	No assoc.
Bezamat et al. (2021); Brazil/Turkey	Cross-sectional	1065 (6–14 y)	EAPD	NR	Assoc. w/meds < 3 y (p = 0.0002)
Fonseca-Souza et al. (2021); Brazil	Cross-sectional	784 (8 y)	EAPD	Questionnaire	Meds assoc. w/MIH (PR = 2.18)
Sulaimani et al. (2021); Saudi Arabia	Cross-sectional	711 (7–9 y)	EAPD	Questionnaire	No assoc.
Acosta et al. (2022); Spain	Cross-sectional	79 (6–9 y)	Ghanim et al., 2015	Medical	Assoc. w/infections, antibiotics
Almuallem et al. (2022); Saudi Arabia	Cross-sectional	1562 (8–12 y)	Ghanim et al., 2015	Questionnaire	Assoc. w/tonsillitis, distress
Mariam et al. (2022); India	Case–control	315 (8–12 y)	EAPD	Questionnaire	Assoc. w/tonsillitis, bronchitis, fever

*MIH* molar-incisor hypomineralization, *mod. DDE* Modified Developmental Defects of Enamel Index, *EAPD* European Academy of Paediatric Dentistry Criteria, *NR* not Reported, *Q.* questionnaire, *Medical* medical diagnosis, *OR* odds ratio, *PR* prevalence ratio, *inf.* infection, assoc. *w/* associated with, *resp*. respiratory

### Risk of bias in included studies

**Fig. 2:**
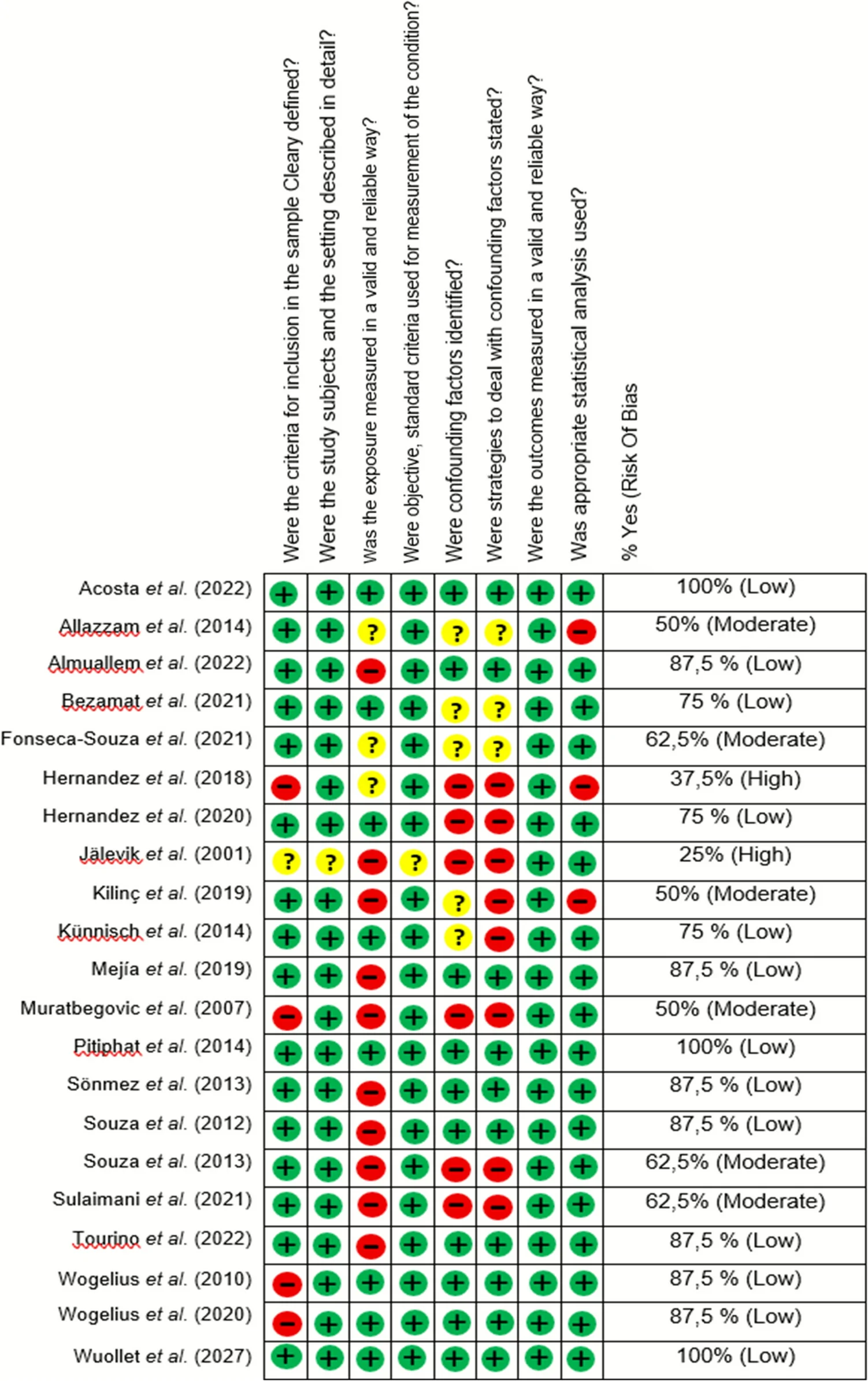
Risk of bias assessment for cross-sectional studies using the Newcastle–Ottawa Scale

**Fig. 3:**
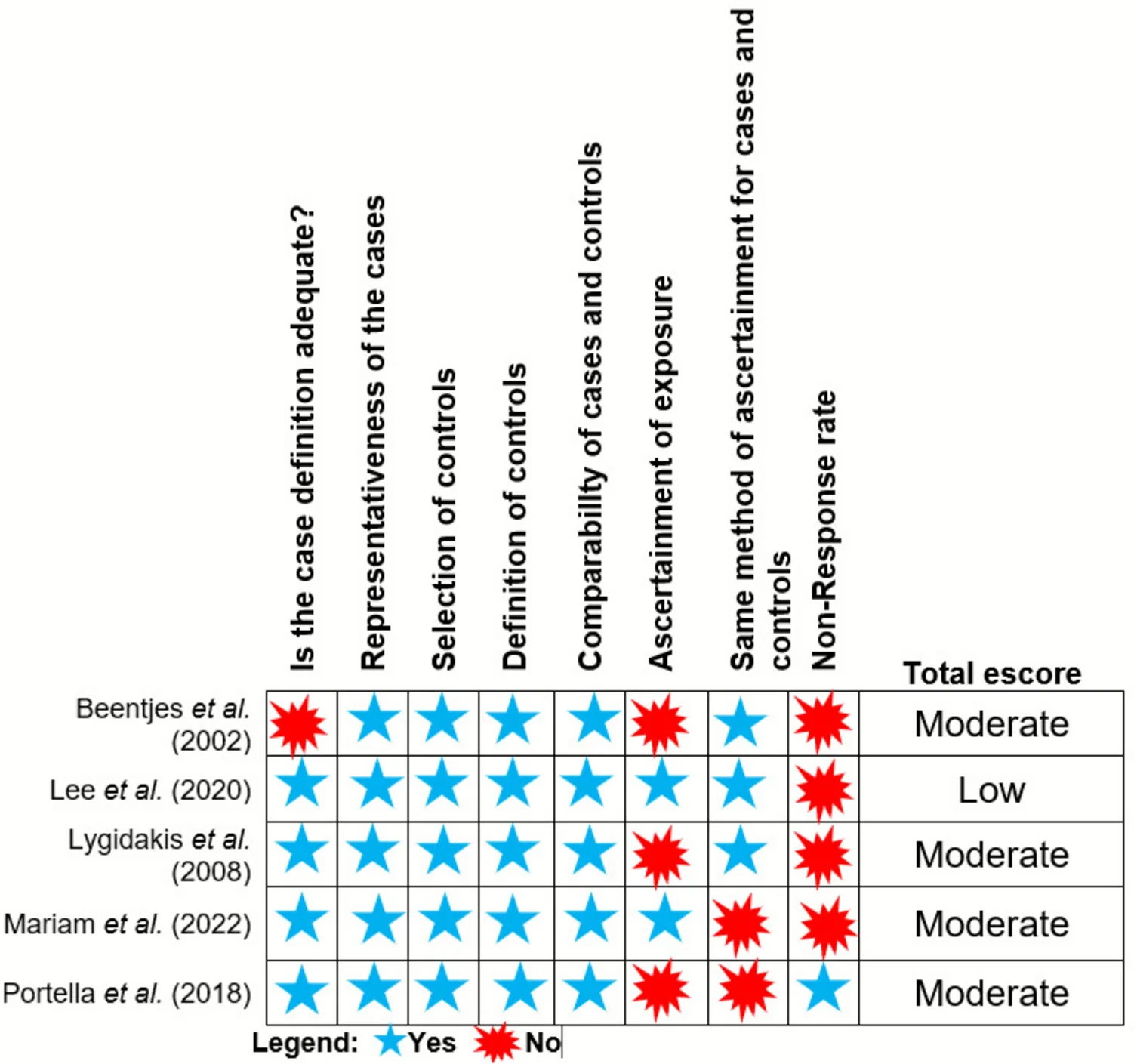
Risk of bias assessment for case–control studies using the Newcastle–Ottawa Scale

**Fig. 4:**
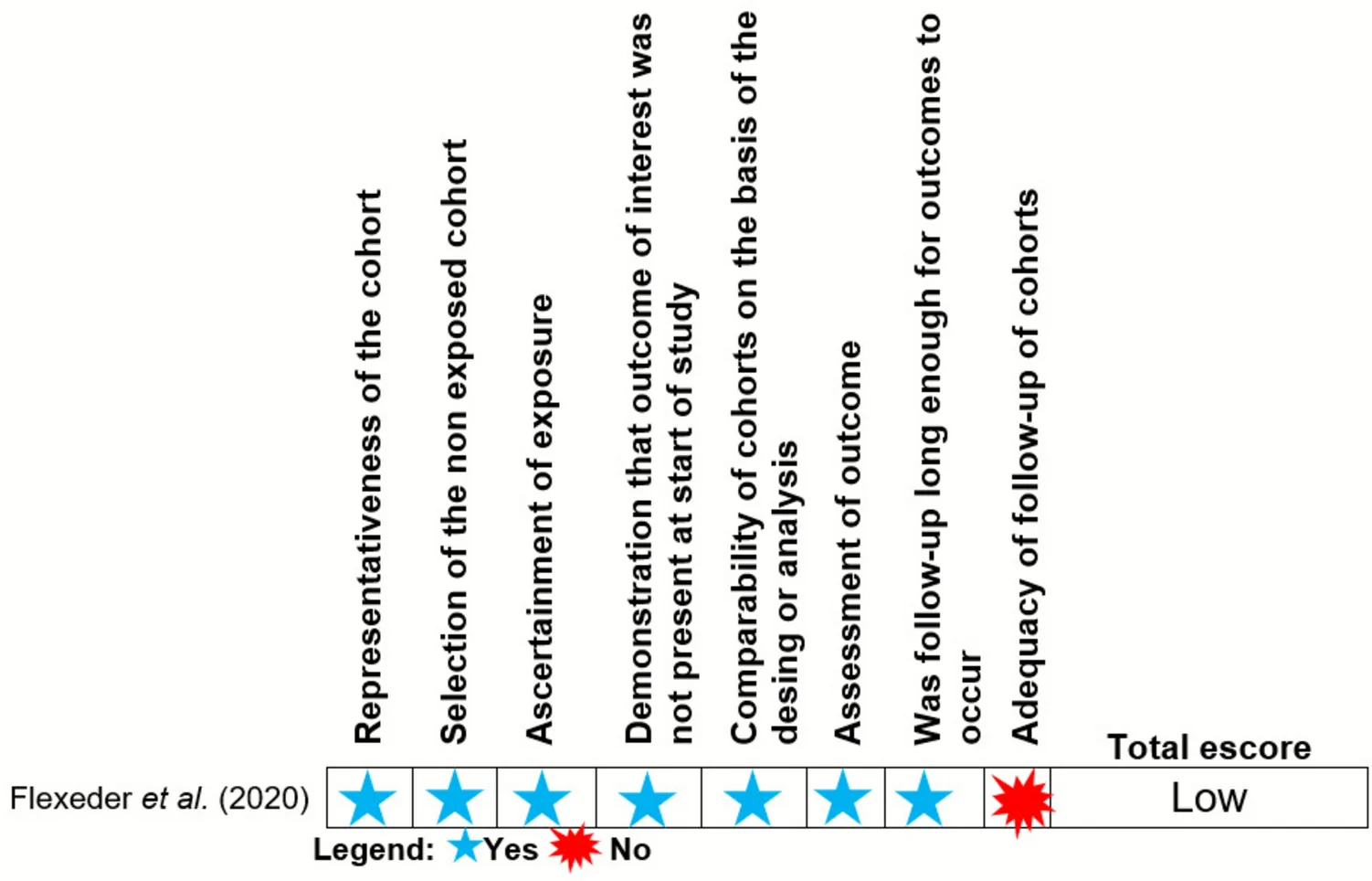
Risk of bias assessment for the cohort study using the Newcastle–Ottawa Scale

The risk of bias assessment for the included studies is presented in Figs. 2, 3 and 4. Most cross-sectional studies demonstrated a low risk of bias (Fig. 2), while the single cohort study also exhibited a low risk (Fig. 4). In contrast, the case–control studies were judged to have a moderate risk of bias (Fig. 3). In cohort studies, the primary limitation was related to the adequacy of study follow-up. For case–control studies, the main methodological concerns were related to the measurement of exposure and the non-response rate, both of which substantially affected study quality.

### Meta-analyses

To quantitatively synthesize the evidence regarding the association between respiratory diseases and molar-incisor hypomineralization (MIH), meta-analyses were performed using both crude and adjusted data. Quantitative synthesis was performed only for outcomes with sufficient data for pooling. Meta-analyses were therefore conducted for asthma and pneumonia. The results are presented in two stages: unadjusted (crude) associations, followed by adjusted estimates that consider potential confounding factors. Pooled estimates were calculated separately according to study design, including case–control and cross-sectional studies.

#### Crude and adjusted analyses

**Fig. 5:**
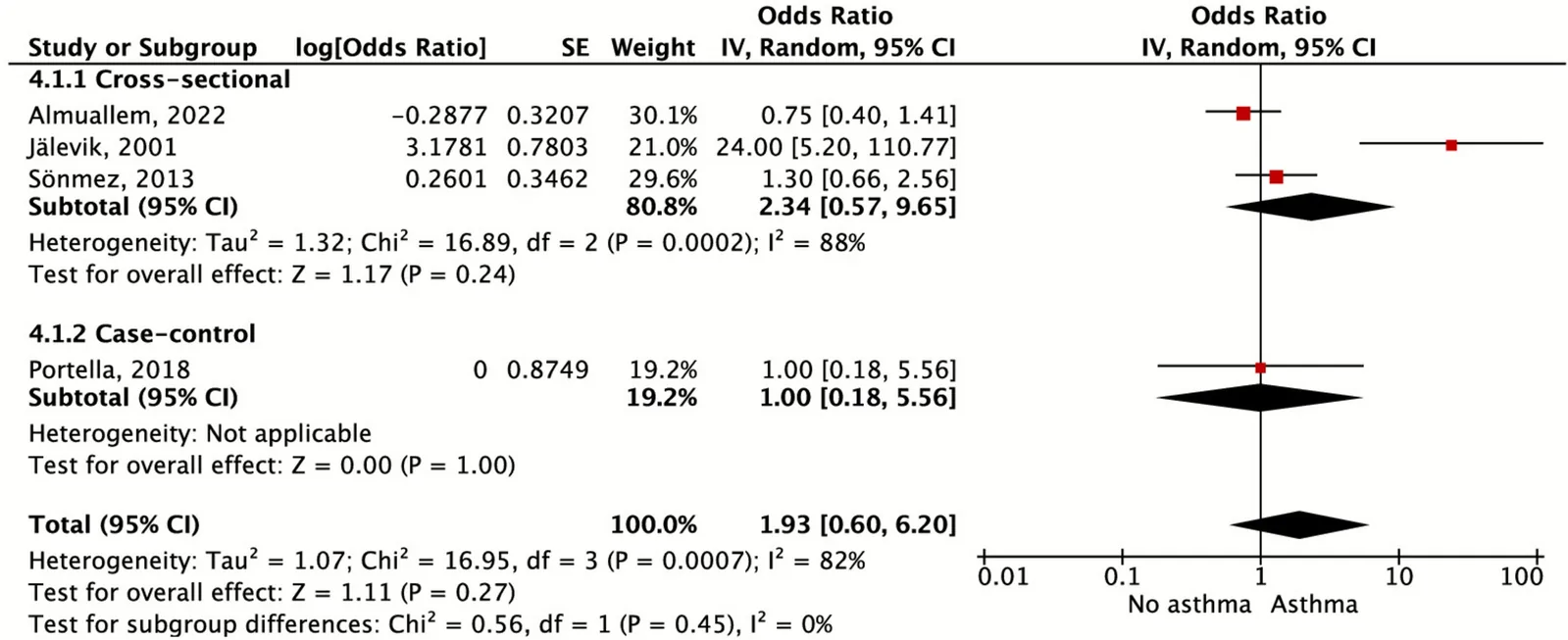
Forest plot: association between asthma and MIH (adjusted analysis). Forest plots present the pooled estimates derived from random-effects models. Estimates are stratified by study design (case–control and cross-sectional studies)

**Fig. 6:**
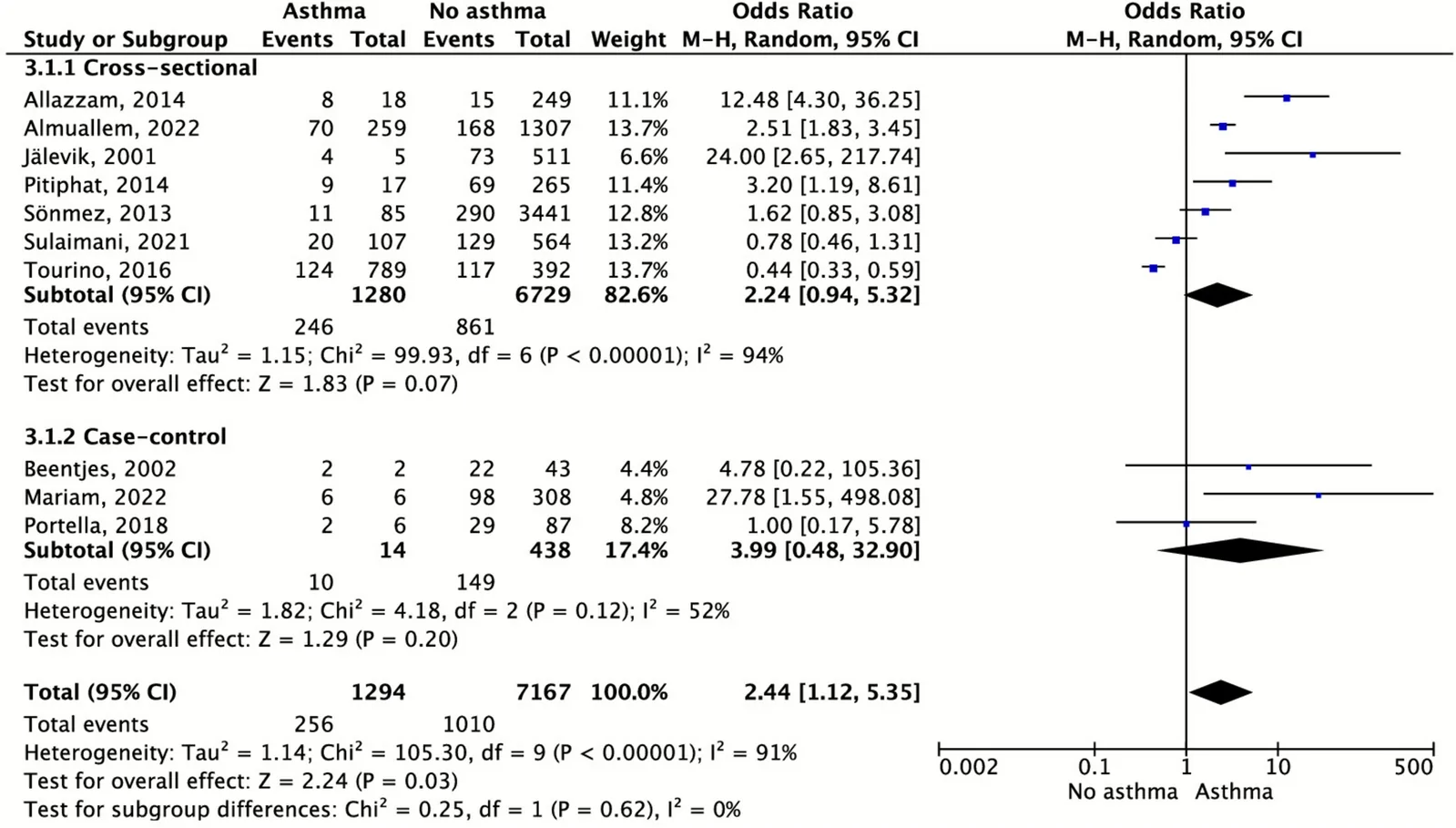
Forest plot: association between asthma and MIH (crude analysis). Estimates are stratified by study design (case–control and cross-sectional studies)

In the adjusted analysis of asthma and MIH, four studies were included [19, 30, 33, 43]. No statistically significant association was observed (OR 1.93; 95% CI 0.60–6.20; I^2^ = 82%; random-effects model) (Fig. 5), although the heterogeneity was high. Moreover, subgroup analyses stratified by study design (case–control and cross-sectional) are presented in Fig. 5 (adjusted) and Fig. 6 (crude). Associations were non-significant within subgroups.

**Fig. 7:**
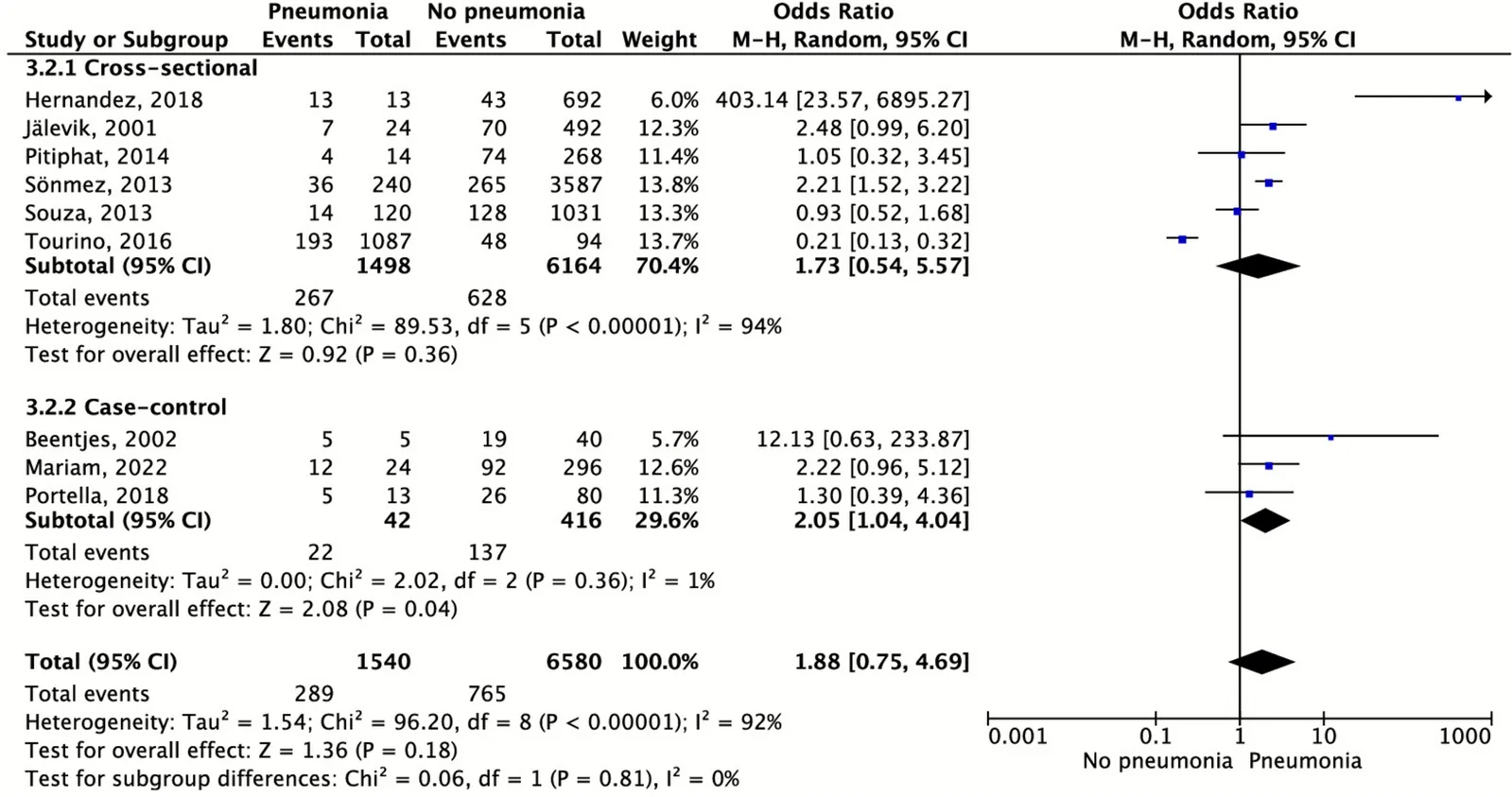
Forest plot: association between pneumonia and MIH (crude analysis). Estimates are stratified by study design (case–control and cross-sectional studies)

**Fig. 8:**
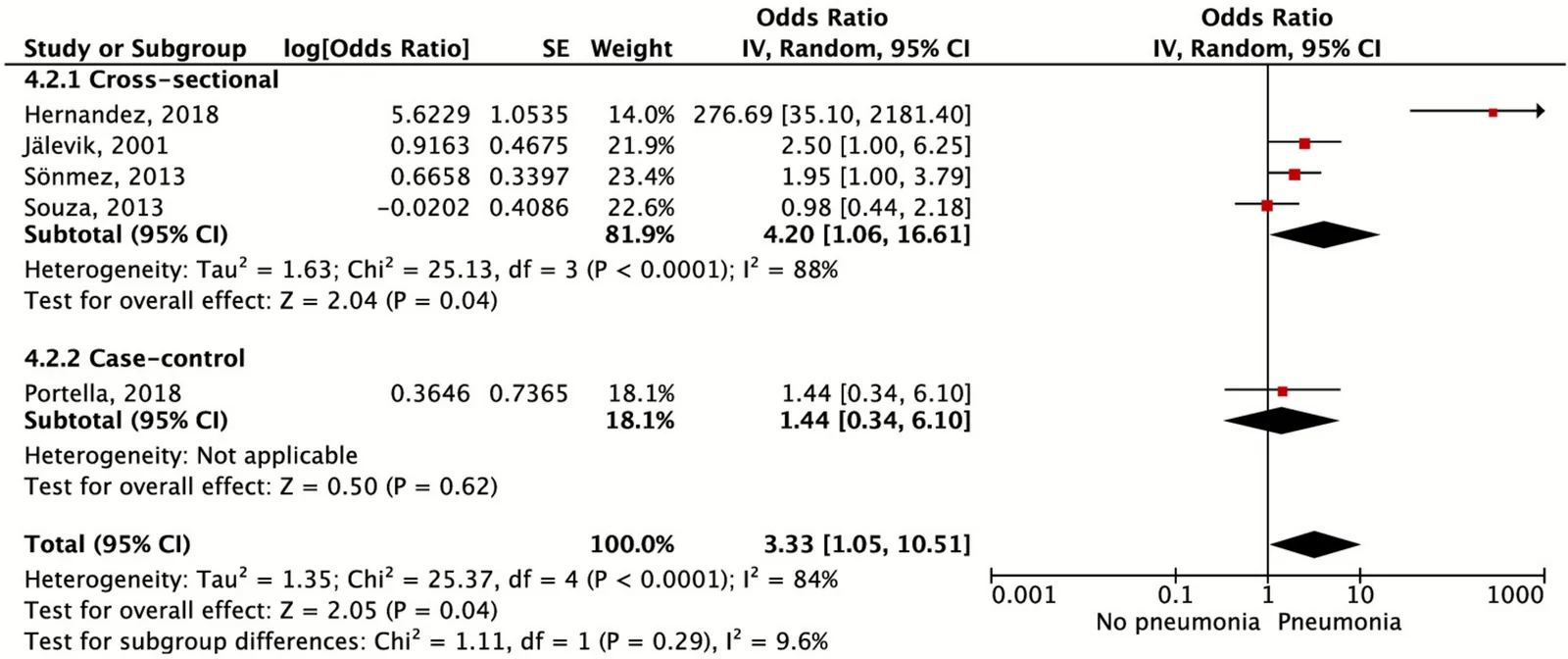
Forest plot: association between pneumonia and MIH (adjusted analysis). Estimates are stratified by study design (case–control and cross-sectional studies)

For pneumonia, five studies were pooled in the adjusted analysis [17, 19, 29, 30, 33]. Meta-analyses were stratified by study design, generating separate estimates for case–control and cross-sectional studies (Figs. 7 and 8, crude and adjusted). A statistically significant association with MIH was observed only in specific subgroups, case–control studies for crude estimates and cross-sectional studies for adjusted estimates, while other subgroups showed non-significant associations. The overall pooled estimates (OR 3.33; 95% CI 1.05–10.51; I^2^ = 84%; random-effects model) should be interpreted cautiously due to substantial heterogeneity and limited power in subgroup analyses, reflecting an association rather than evidence of causality.

### Results of individual studies

The associations between MIH and tonsillitis [30, 43, 47], bronchitis [30, 47], upper and lower respiratory tract infections [19, 20], and non-specific respiratory conditions [12, 32] were each evaluated by a limited number of studies. Given the small number of eligible studies per outcome and the inconsistency of findings across reports, quantitative pooling was deemed inappropriate. These outcomes were therefore synthesized narratively.

In this sense, three studies assessed the relationship between tonsillitis and MIH [30, 43, 47]. In a Brazilian case–control study of 93 children aged 6–13 years, Portella et al. [30] reported no significant association between MIH and respiratory conditions, including tonsillitis. Conversely, Almuallem et al. [43], in a large cross-sectional study conducted in Saudi Arabia (n = 1562; aged 8–12 years), observed strong associations between MIH and both respiratory distress (OR 5.98; 95% CI 9.34–20.16) and tonsillitis (OR 12.65; 95% CI 9.12–44.62). Similarly, Mariam et al. (2022), in a case–control study from India involving 315 children aged 8–12 years, identified significant associations between MIH and tonsillitis (OR 3.00; 95% CI 1.17–7.70), as well as bronchitis, fever, and other early childhood illnesses [47]. Collectively, these findings suggest that geographic, methodological, or contextual factors may influence the observed associations.

Two case–control studies specifically examined bronchitis in relation to MIH [30, 47]. While Portella et al. [30] reported no significant association (OR 1.50; 95% CI 0.58–3.91), Mariam et al. (2022) found a positive association (OR 2.57; 95% CI 1.34–4.90) [47]. These discrepancies may reflect differences in sample size, population characteristics, or exposure ascertainment, highlighting the need for standardized methodologies in future research.

Upper and lower respiratory tract infections were evaluated in two cross-sectional studies [19, 20]. Sönmez et al. (2013), in a Turkish cohort of 4,049 children aged 7–12 years, reported significant associations between MIH and pneumonia (p = 0.001) and fever (p = 0.007), but not with asthma or general respiratory tract infections [19]. In contrast, Wuollet et al. [20], studying 287 Finnish children aged 7–12 years, identified significant associations between MIH and acute otitis media, as well as early childhood antibiotic use, including penicillins, amoxicillin, and macrolides.

Finally, two cross-sectional studies explored broader indicators of respiratory morbidity in early childhood [12, 32]. Mejía et al. [12], in a Colombian sample of 1075 children aged 6–15 years, found a significant association between MIH and a history of early childhood respiratory problems (OR 2.48; 95% CI 1.63–3.78). Similarly, Fonseca-Souza et al. [32], analyzing 784 Brazilian children aged 8 years, reported that medication use during early childhood—used as a proxy for systemic conditions such as respiratory diseases—was significantly associated with MIH (PR 2.18; 95% CI 1.06–4.48).

### Certainty of evidence

Based on the available evidence, the certainty of the findings regarding the associations between MIH and respiratory conditions, whether derived from quantitative synthesis or narrative assessment, was judged to be very low, as shown in Table 2. Several outcomes were characterized by extremely wide confidence intervals, indicating substantial imprecision and, therefore, limiting confidence in the magnitude and direction of the observed effects.

**Table 2:** Certainty of evidence of included studies according to GRADE

Certainty assessment							№ of patients		Effect		Certainty	Importance
№ of studies	Study design	Risk of bias	Inconsistency	Indirectness	Imprecision	Other considerations	Respiratory problems	no respiratory problem	Relative (95% CI)	Absolute (95% CI)		
MIH and asthma—crude data												
10	Non-randomised studies	Serious^a^	Very serious^b^	Not serious	Serious^c^	Strong association^d^	256/1294 (19.8%)	1010/7167 (14.1%)	OR 2.44 (1.12–5.35)	145 more per 1000 (from 14 to 326 more)	⨁◯◯◯ Very low^a,b,c,d^	Critical
MIH and asthma—adjusted data												
4	Non-randomised studies	Serious^e^	Very serious^f^	Not serious	Very serious^g^	None	–	–	OR 1.93 (0.60–6.20)	2 fewer per 1000 (from 6 to 1 fewer)	⨁◯◯◯ Very low^e,f,g^	Critical
MIH and pneumonia—crude data												
9	Non-randomised studies	Serious^h^	Very serious^i^	Not serious	Very serious^g^	None	289/1540 (18.8%)	765/6580 (11.6%)	OR 1.88 (0.75–4.69)	82 more per 1000 (from 26 fewer to 265 more)	⨁◯◯◯ Very low^g,h,i^	Critical
MIH and pneumonia—adjusted data												
5	Non-randomised studies	Serious^j^	Very serious^k^	Not serious	Very serious^c^	Strong association^l^	–	–	OR 3.33 (1.05–10.51)	3 fewer per 1000 (from 11 to 1 fewer)	⨁◯◯◯ Very low^c,j,k,l^	Critical

Incidence of MIH = (number of patients with MIH/total number of patients with or without respiratory problems) × 100

*CI* confidence interval, *OR* odds ratio

Explanations

^a^Risk of bias was detected in 5 studies, representing 40.1% for the overall analysis

^b^Very high heterogeneity was identified (I^2^ = 91%; p < 0.001)

^c^A wide 95% confidence interval was identified for the overall estimate

^d^As the overall odds ratio was 2.44, this is considered a large effect

^e^Risk of bias was detected in 1 study, representing 21% for the overall analysis

^f^Very high heterogeneity was identified (I^2^ = 82%; p < 0.001)

^g^No statistically significant association was detected. A very wide 95% confidence interval was observed

^h^Risk of bias was detected in 5 studies, representing 49.9% for the overall analysis

^i^Very high heterogeneity was identified (I^2^ = 92%; p < 0.001)

^j^Risk of bias was detected in 3 studies, representing 58.5% for the overall analysis

^k^Very high heterogeneity was identified (I^2^ = 84%; p < 0.001)

^l^As the overall odds ratio was 3.33, this is considered a large effect

## Discussion

This systematic review is the first to investigate the association between early childhood respiratory problems and molar incisor hypomineralization (MIH). Despite the considerable heterogeneity among the included studies, the analyses suggested possible associations between MIH and certain respiratory conditions. However, these findings must be interpreted cautiously given the very low certainty of the available evidence.

Respiratory diseases rank among the most prevalent conditions affecting children in the early years of life (birth to 3 years of age) and have been extensively investigated in the literature [40, 45, 49]. In contrast to previous studies that reported no association between MIH and respiratory conditions [29–32, 34, 35, 40–42], several of the studies included in this review identified such association [4, 8, 12, 17, 19, 20, 28, 33, 36, 37, 39, 43–48]. Furthermore, associations were reported between MIH and specific respiratory infections, including asthma [4, 8, 19, 30, 33, 36, 41, 43, 48], as well as various upper and lower respiratory tract infections [8, 17, 19, 20, 28–30, 33, 36, 37, 41, 42, 44, 47, 48]. 

Asthma, a chronic obstructive inflammatory disorder, has a high incidence and prevalence, contributing to approximately 2.5 million deaths annually [50, 51]. Hypoventilation resulting from airway obstruction—either by mucous hypersecretion or inflammation-induced edema—triggers a cascade of physiological responses, including carbon dioxide accumulation, respiratory acidosis, and hypoxia [14, 15, 51], which may potentially interfere with amelogenesis [15, 37].

During the secretory stage of amelogenesis, enamel matrix proteins such as amelogenin, ameloblastin, and enamelin are synthesized and secreted [52]. In the subsequent maturation phase, the enamel crystals increase in size as these proteins are degraded, removed, and replaced by hydroxyapatite-based inorganic material [52]. Ameloblasts are highly sensitive cells, and any disturbance—such as pH alterations induced by respiratory acidosis—can inhibit the activity of proteolytic enzymes [15, 52]. This disruption may lead to prolonged retention of matrix proteins, interfering with the progressive growth of hydroxyapatite crystals and resulting in enamel that is hypomineralized, porous, and structurally compromised—hallmarks of MIH [15, 52].

The pharmacological management of asthma typically involves inhaled corticosteroids or long-acting β-agonists, depending on the disease phenotype and origin [50]. Prior studies have suggested that glucocorticoids and corticosteroids may impair bone development by inducing osteoblast apoptosis [53]. Consequently, a similar deleterious effect on ameloblasts may be hypothesized, potentially contributing to the formation of hypomineralized lesions. However, in this review, most studies assessing asthma therapies did not find a significant association between MIH and asthma medications [35, 40, 45]. Therefore, it is plausible that the disease itself, particularly when inadequately controlled, manifesting as hypoxia, plays a more prominent role in disrupting enamel development than the pharmacological treatment.

Common pediatric infections such as bronchitis, pneumonia, acute otitis media, and tonsillitis frequently induce febrile episodes [30, 39, 43]. Although fever is an adaptive response that aids recovery, it has been implicated as a contributing factor to MIH [43, 47]. Experimental studies in animal models have demonstrated microstructural enamel disruptions following febrile episodes [15, 54], as well as alterations in the expression of genes involved in essential cellular processes under elevated body temperatures [54].

Polymorphisms in genes associated with immune regulation, such as TGF and TGFA [31], and those involved in adaptive responses to hypoxia [49], have also been proposed as potential contributors to hypomineralization. Given the central role of TGF and TGFA genes in modulating inflammation during early childhood respiratory illnesses, genetic variations may indirectly affect amelogenesis and contribute to the development of MIH. Additionally, the HIF-1 gene (Hypoxia-Induced Factor-1), which regulates tissue responses to hypoxia by enhancing oxygen delivery and promoting adaptive metabolism, has been implicated in this context [55]. The strong link between respiratory illness and hypoxia suggests that mutations in this gene may further predispose to enamel hypomineralization. However, further investigations are necessary to substantiate this potential genetic association.

Hypocalcemia has also been identified as a possible etiological factor for MIH [33, 56] and has been associated with airway infections caused by pathogens such as *Neisseria meningitidis* [33]. Reduced calcium content in hypomineralized enamel has been documented in both in vitro studies and clinical investigations involving children hospitalized with respiratory infections. Consequently, it is plausible that diminished bioavailable calcium during the maturation stage of amelogenesis contributes to hypomineralization. Supporting this hypothesis, immunocytochemical assays in murine models have shown that hypocalcemia impairs the breakdown and removal of enamel proteins, altering hydroxyapatite prism organization during enamel maturation [33, 57].

Regarding pharmacological exposure, among the 11 studies evaluating the association between medication use and MIH [4, 8, 12, 20, 29, 32, 36, 40–43], five studies [20, 29, 32, 36, 42] identified drug intake as a potential etiological factor. Amoxicillin was the most frequently cited, likely due to its widespread use and favorable safety profile. Nevertheless, although an association between amoxicillin use and MIH was observed [20, 29, 32, 36, 42], these findings should be interpreted cautiously. The study designs employed in most cases did not support causal inferences, and it remains unclear whether the observed dental defects were attributable to the medication exposure, the underlying illnesses, or residual confounding inherent to observational designs.

Notably, although some individual studies suggested potential links between MIH and various respiratory illnesses, the inconsistency in outcome measures, exposure definitions, and study designs limited the strength of the evidence. The lack of adjustment for potential confounders in many of the included studies, combined with a predominance of cross-sectional designs, contributes to the overall very low certainty in the observed associations.

To the best of our knowledge, no other review has systematically integrated the existing evidence regarding diverse respiratory conditions described in the literature, providing a thorough perspective on their potential contribution to the etiology of MIH. Recognizing these associations may aid in the early detection of risk factors and support the formulation of targeted public health interventions aimed at preventing systemic conditions that may disrupt enamel formation. Additionally, the results highlight the critical need for interdisciplinary collaboration between healthcare professionals, particularly physicians and dentists, to ensure comprehensive care for pediatric patients.

Nevertheless, the findings of this review should be interpreted with caution. Most included studies utilized cross-sectional designs, which inherently limit conclusions regarding causality and temporality. Substantial heterogeneity across studies precluded meta-analysis for certain outcomes and hindered direct comparisons. This heterogeneity reflects variability in exposure assessment, disease definitions, outcome diagnosis, age ranges, and confounder adjustment strategies, collectively undermining the precision of pooled estimates. Further subgroup analyses by respiratory diagnosis, data source or disease severity were not conducted due to the limited number of available studies within each category, which would have resulted in statistically underpowered subgroup estimates. Several pooled estimates exhibited wide confidence intervals, reflecting substantial statistical imprecision and further limiting clinical interpretation.

Additionally, reliance on retrospective recall questionnaires to assess exposures during early childhood introduces potential recall bias, especially given the considerable time lapse between exposure and data collection. The lack of standardized and adequately completed medical records also compromised the reliability of exposure assessment. Moreover, outcomes supported by only two or three studies were not meta-analyzed due to concerns regarding the reliability of pooled estimates under conditions of extreme heterogeneity and sparse data and were therefore interpreted narratively.

Finally, most included studies were conducted in Brazil, which may limit the generalizability of the findings to countries with different healthcare systems, clinical practices, and population characteristics. Notably, the included evidence was not exclusively restricted to Brazil, as studies from Saudi Arabia, Spain, Turkey, and other regions were also identified. Nevertheless, further well-designed studies conducted in diverse international settings are needed to strengthen the robustness and global applicability of the conclusions.

## Conclusion

This systematic review suggests a possible association between early childhood respiratory conditions and molar–incisor hypomineralization (MIH), particularly for pneumonia. However, evidence from adjusted meta-analyses was inconsistent, and no significant association was confirmed for asthma. For several outcomes, quantitative synthesis was not feasible due to limited and heterogeneous data, requiring narrative interpretation. Therefore, the associations observed should be interpreted with caution due to the substantial heterogeneity across studies and the predominantly very low certainty of evidence according to the GRADE framework. Current findings indicate a possible association but do not support definitive causal inference. Prospective cohort studies following children from birth through early childhood and the development of their permanent dentition are warranted to establish causal relationships and minimize recall bias. Moreover, the standardization of diagnostic criteria for respiratory conditions is essential to facilitate robust data synthesis and accurate comparisons across studies.

## Supplementary Information

Below is the link to the electronic supplementary material.

Supplementary file1 (DOCX 30 KB)

## Data Availability

The datasets generated and analyzed during the current study are available from the corresponding author upon reasonable request.
